# Spatiotemporal dynamics and associated drivers of COVID-19 incidence in Nepal

**DOI:** 10.1186/s41182-025-00741-5

**Published:** 2025-08-19

**Authors:** Bipin Kumar Acharya, Shristi Sharma, Laxman Khanal, Pramod Joshi, Meghnath Dhimal

**Affiliations:** 1 Planetary Health Research Centre, Kathmandu, Nepal; 2 Nepal Geographical Society, Kathmandu, Nepal; 3 Western University, Richmond St, London, Canada; 4 Central Department of Zoology, Institute of Science and Technology, Tribhuvan University, Kathmandu, 44618 Nepal; 5 International Centre for Biodiversity and Primates Conservation, Dali University, Dali, 671003 Yunnan China; 6 Nepal Health Research Council, Kathmandu, Nepal

**Keywords:** Epidemic hotspots, GeoDetector, Incidence rate, Spatial statistics, Targeted intervention

## Abstract

**Background:**

COVID-19 has been a major global health concern, severely impacting Nepal with thousands of cases and deaths. The patterns of COVID-19 incidence in the country may have varied over time during the pandemic, with geographic factors playing different roles in the early, middle, and later phases of transmission.

**Methods:**

We utilized spatial statistics and GeoDetector methods to analyze district-level variations in COVID-19 incidence across Nepal from January 2020 to December 2022 using laboratory confirmed cases of the disease and a range of physical, biological and socioenvironmental explanatory variables. The analysis focused on identifying spatial patterns, hotspots, and key driving factors contributing to the uneven distribution of COVID-19 cases.

**Results:**

We found an uneven distribution of COVID-19 in Nepal, with persistent hotspots in major cities, such as Kathmandu and Pokhara, reaching up to 133 cases per 1000 population. GeoDetector analysis identified the key drivers, including road density (q = 0.59, p < 0.001), ICU bed distribution (q = 0.51, p < 0.001), and population density (q = 0.46, p < 0.001). While natural environmental factors such as temperature, precipitation, and NDVI had low and statistically insignificant independent explanatory power, their interaction with variables such as nighttime light, NDVI, and population density enhanced explanatory strength, highlighting the complex spatial distribution of COVID-19 incidence.

**Conclusions:**

We recommend that the Nepalese government implement more targeted and region-specific interventions to address COVID-19 outbreaks, especially in persistent hotspot areas, such as Kathmandu and other emerging cities.

## Introduction

The novel coronavirus disease 2019 (COVID-19) caused by the severe acute respiratory syndrome coronavirus 2 (SARS-CoV-2) first identified in Wuhan, China in late December 2019 [1] became a pandemic and global public health crisis to the mankind of this century. Due to its high contagiousness, COVID-19 spread quickly despite several efforts to contain the transmission. On March 11, 2020, the World Health Organization proclaimed a pandemic of global significance, requiring the entire world’s attention to control the disease, lower mortality rates, and stop its spread [2]. The successful development and administration of vaccines against SARS-CoV-2 have significantly reduced cases, prompting the WHO to lift the “international public health emergency” [3]. Life has largely returned to normal with restrictions on movement been lifted. However, the threat of new variants and potential outbreaks persists, underscoring the need to understand transmission dynamics and lessons from the pandemic to prepare for future outbreaks.

The first COVID-19 case in Nepal was reported on January 23, 2020 [4]. Following that, the Nepal government put in place several restrictive measures, including lockdowns and limitations on movement, to slow and manage the pandemic’s spread. However, incidence and death rates increased across Nepal and experienced three separate waves [5]. The first wave of COVID-19 began in June 2020, peaked in late October 2020, and began to progressively decline around January 2021 [6]. The second wave, which was mostly caused by the delta variant, happened between March and June of 2021, coinciding with a wave of a similar nature that occurred in India [5]. Following the second wave, fewer instances were reported in December 2021, indicating a considerable decline in the cases. However, the first Omicron form of COVID-19 was discovered on December 6, 2021, which caused the number of cases to rise once again, later peaked in January 2022 and subsided in February 2022 resulting in the third wave [6]. The interplay of complex geographical features of Nepal alongside rural–urban gaps in population dynamics and the socio-economic spectrum formed an intricate system of managing the pandemic [7]. This was made even more complex due to the unrestricted cross border movement with India [8].

Spatial heterogeneities of infectious diseases can arise from a variety of factors. These include intrinsic population processes, such as the spatial aggregation of infected individuals and their nonrandom social interactions, as well as environmental influences that vary across different spatial locations [9, 10]. In addition, the spatial interconnectedness of regions, where the movement and interaction of people across different areas impact the spread of the disease, plays a significant role [11, 12]. Previous studies have shown tremendous spatial variations and heterogeneity in the distribution of COVID-19 incidence across the world in different spatiotemporal scales [13–15].

Geographic information system (GIS) is an essential tool to examine the spatial distribution of infectious diseases [16]. Several GIS-based studies have been published, since the initial outbreak of COVID-19 [15, 17, 18] which have recognized spatial pattern as an essential dimension of disease processes [19] suggesting understanding spatial distribution patterns is essential for its mitigation, as it helps to clarify the extent and impact of the pandemic and can aid decision making, planning and community action [20]. In addition, knowing the variables/drivers that affect the spatiotemporal variation of the distribution and the mechanism how they affect are also important. A few studies were conducted to identify those factors with intriguing results. According to Ma et al. [21] humidity has a negative association with deaths caused by COVID-19. COVID-19 diffusion has been found highly correlated with city air pollution measured on days when PM10 (particulate matter with a diameter of less than 10 μm) or ozone levels exceed established limits [22]. The findings show that the mechanisms of air pollution-to-human transmission are more important than those of human-to-human transmission [23]. However, these results should be interpreted with high caution as the environmental factors may have different roles during the different phases of the pandemic. For example, the role of temperature and humidity was strong in the early phase of the outbreak but became weaker later [24]. In addition, inequalities in geographic accessibility to health care can influence the spatiotemporal variation of disease distribution. Travel time to health service providers appears to have a detrimental influence on healthcare-seeking behavior, as evidenced by the established unfavorable health outcomes associated with seasonal influenza transmission and other diseases in the US caused by disparities in geographic accessibility to healthcare [25]. In addition, the actual number of cases could be larger than the reported numbers as the disease could likely have gone undetected in most asymptomatic cases [5].

Despite its unprecedented severity, limited studies have been conducted on COVID-19 in Nepal from spatial perspectives. For example, a study visualizes in district, provincial, and district level aggregated COVID-19 cases data till January 2021 [26] while another study assessed the spatiotemporal dynamics of COVID-19 in the context of various restrictions imposed to contain the disease transmission [7]. Studies incorporating high resolution spatial data are lacking in low- and middle-income countries including Nepal mainly due to the restricted availability of reliable data. For example, while geographically weighted regression (GWR) has been effectively used in other contexts to analyze COVID-19 incidence rates and reveal spatial variations in disease spread [27], similar approaches in Nepal are lacking due to data limitations. In addition, GeoDetector-based methods have been proven effective to identify how different factors influence COVID-19 distribution [28]. Time-varying factor GWR and multi-scale geographically weighted regression (MGWR) have been used to capture changes in disease patterns over time [14], which have not been tested yet in Nepal. Spatiotemporal distribution of COVID-19 incidences, changes in the spatial distribution over time, drivers associated with the spatial process and whether the effect of these drivers changes over time are not known so far, highlighting the urgent need for more comprehensive spatial analyses.

The spatial distribution patterns of COVID-19 incidence may have changed in different dates during the pandemic and role of geographic factors may be different in early, mid or later phases of the transmission [24]. Difficult mountainous landscape and remote terrain, unrestricted movement across open border with India, under-resourced health system facility, poverty and inequality, political instability leading to policy shifts, etc. might have contributed in COVID-19 spread across Nepal. We hypothesized that the COVID-19 incidence rates and spreads were higher in the areas of higher population density and mobility. Since mobility is influenced by the complex geography and limited road networks and health facilities of the country, clustering of the COVID-19 incidence might be in and around the mega cities [29]. Therefore, the aim of this study was to explore the spatial distribution of incidence rate and spreading patterns of COVID-19 in Nepal during the first three waves and assess the influencing factors for the heterogeneous spatial distribution across these waves. The study provides evidence to health policy makers to allocate the limited resources in appropriate time and space.

## Methods

**Fig. 1:**
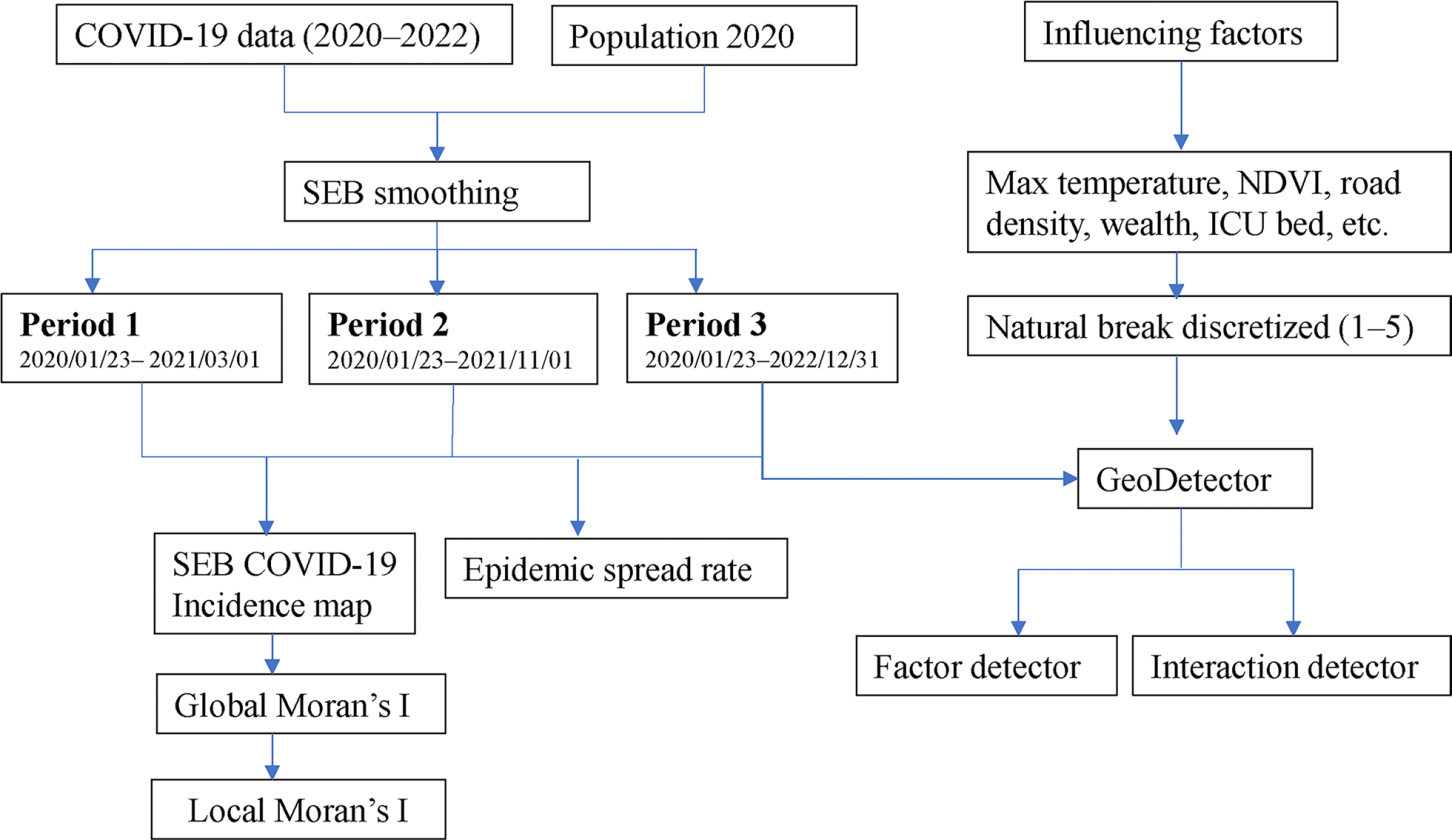
Flowchart showing the methodological workflow of the study

This study employed laboratory confirmed COVID-19 cases in Nepal between January 2020 and December 2022 and a range of explanatory variables to identify the spatiotemporal transmission dynamics of the disease in Nepal. The detailed methodological workflow is presented in Fig. 1.

### COVID-19 data

The laboratory confirmed COVID-19 cases were obtained from the situation reports published by the Ministry of Health and Population (MOHP) of the Government of Nepal which was publicly available from the MOHP web portal (https://mohp.gov.np/np). The data set contains the numbers of confirmed COVID-19 cases across 77 districts of Nepal reported on daily basis until November 2020 and then weekly till December 30, 2022. We extracted the cumulative cases for three different time following the first, second and third waves of COVID-19 in Nepal and characterized the spatiotemporal dynamics for all three waves.

### Explanatory variables

We collected a range of explanatory variables from different open access geoportals comprising physical, biological and social environment that can influence spatial distribution of COVID-19 incidence. The full list of explanatory variables and data sources are listed in Table 1.

**Table 1:** Potential influencing factors of COVID-19 incidence in Nepal

Type	Data sets	Sources	Resolution/unit	Year	Code
Climate and environment	Maximum Temperature	Worldclim	1 km	2020	X1
	Minimum Temperature				X2
	Precipitation				X3
	NDVI	MODIS Terra	1 km	2020	X4
Demography	Population	WorldPop	1 km	2020	X5
	Elderly population				X6
Socioeconomic inequalities	Road density		1 km		X7
	POI density	Open Street Map	Count	2020	X8
	Night-time light	WorldPop	nanoWatts/cm^2^/sr *1E9	2021	X9
	Distribution of Wealth				X10
Distribution of medical resources	ICU bed	DRR Portal	Count	2021	X11
	Ventilators				X12
	Oxygen cylinder				X13

The mean maximum temperature, mean minimum temperature and mean annual precipitation were retrieved from WorldClim geoportal (http://worldclim.org/version2) in 1 km spatial resolution. The WorldClim data set is a product based on the observation recorded from the worldwide distribution monitoring station collected between 1970 and 2000. The detail methodology of creation and validation of these data source has been discussed elsewhere [30]. The mean annual NDVI was extracted from MOD13C25 MODIS product using the Geospatial Interactive Online Visualization and Analysis Infrastructure (GIOVANNI) system in the NASA Goddard Earth Science Data and Information Service Center (GES DISC) (https://giovanni.gsfc.nasa.gov/).

We used two data sets to represent population dynamics, the first one is population density, and another is proportion of elderly people. Both these data sets were downloaded from WorldPop portal (https://hub.worldpop.org/) in 100 m spatial resolution.

**Fig. 2:**
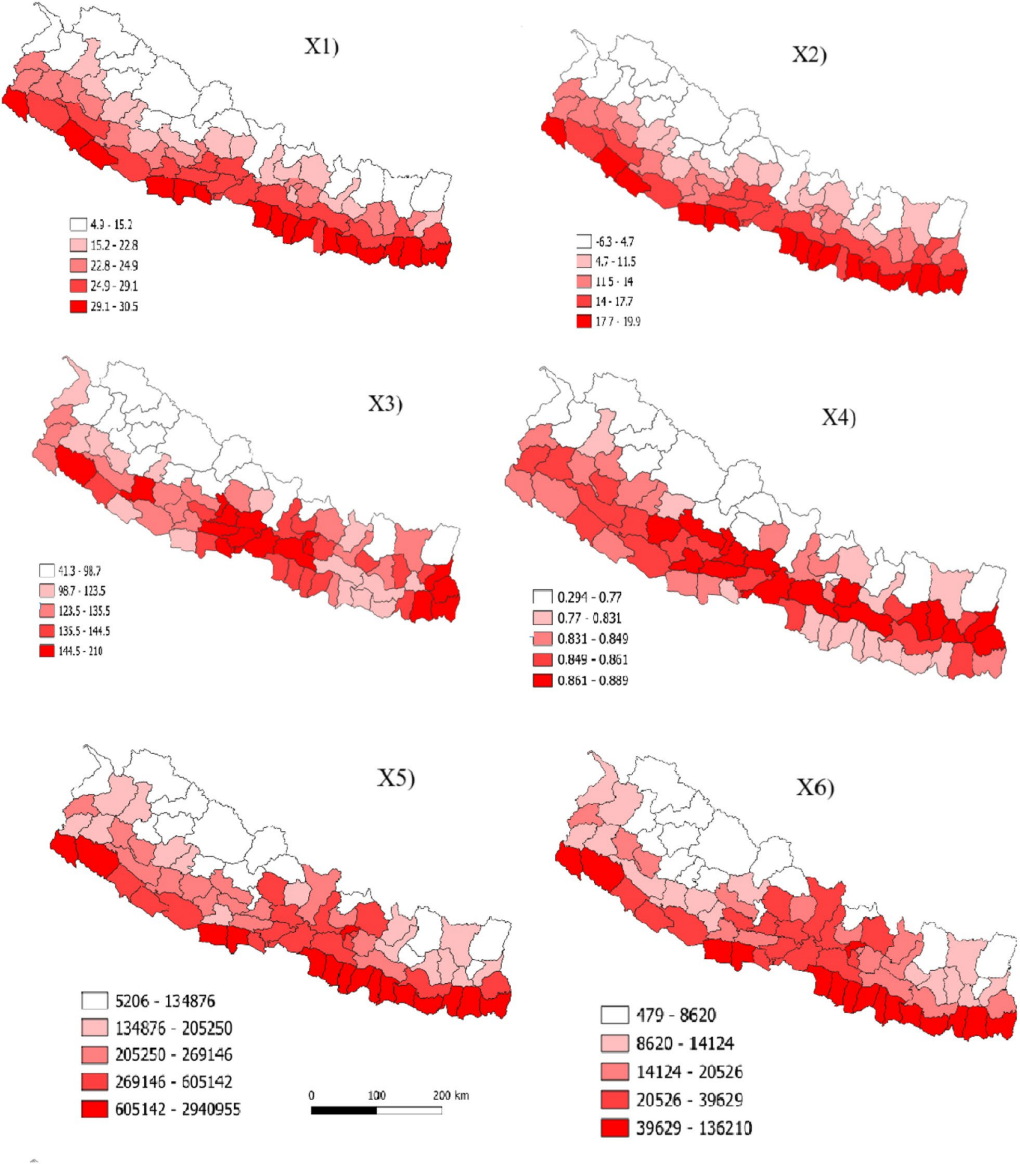

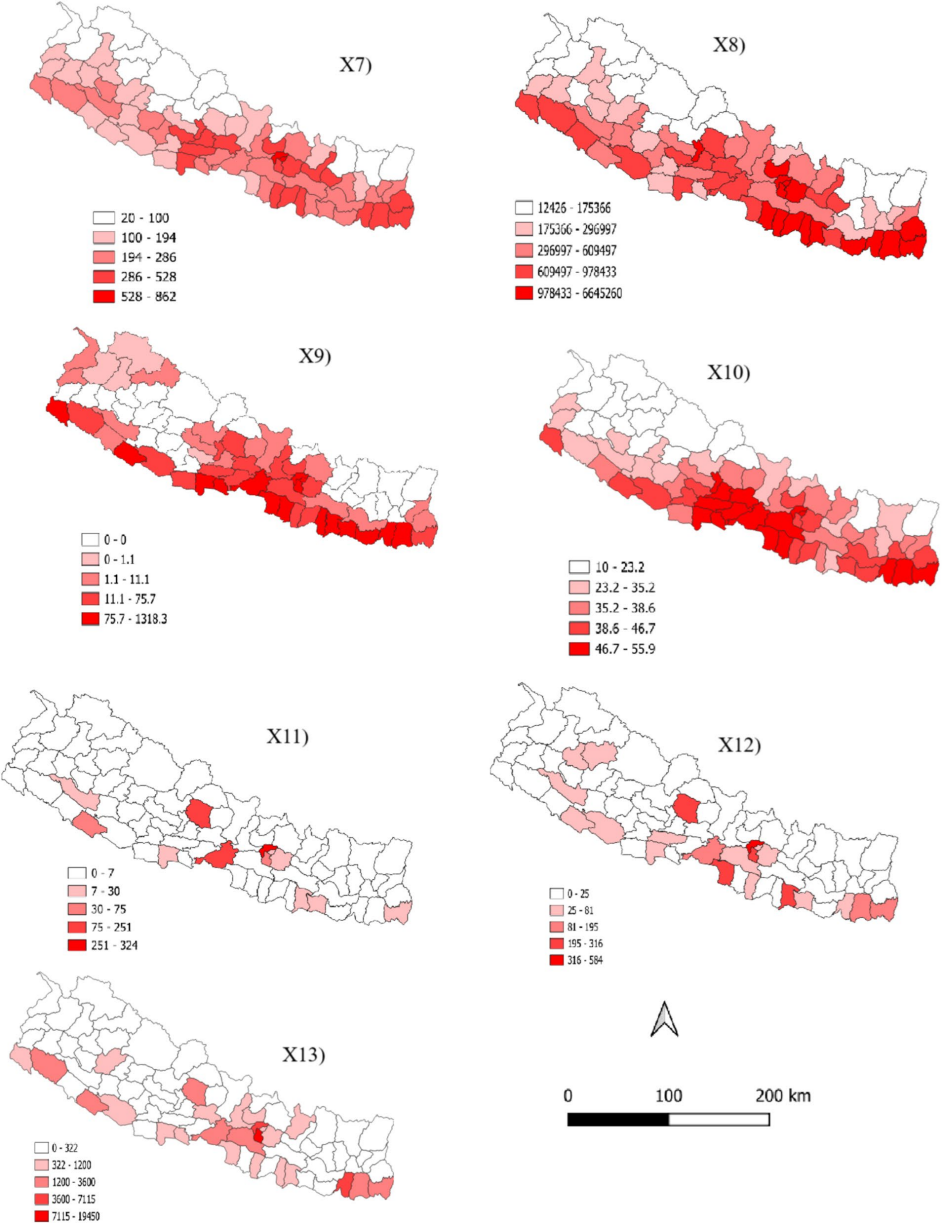
Spatial distribution of 13 selected variables summarized in 77 districts of Nepal. The figure numbers correspond to the codes used for predictor variables in Table 1

To represent the socioeconomic inequalities, we chose five different variables. The first variable of this category was road density which was collected from Global Roads Inventory Project’s global roads database (https://www.globio.info/download-grip-dataset) in 5 arcminutes spatial resolution [31]. Points of interest (POI) was the second variable which was computed based on the POI data sourced from the open street map (https://www.openstreetmap.org/). For night time light (NTL), median product for the year 2020 of the VIIRS Annual Night Time Lights version 2 (VNL v2) data set was used [32]. The NTL is a widely used proxy related to human activities, such as population density, wealth and urbanization. Furthermore, we extracted the distribution of medical resources such as number of ventilators, ICU bed and oxygen cylinder available in the district from Nepal Disaster Risk Reduction Portal (http://drrportal.gov.np/). The explanatory variables measured by continuous data were summarized on the zonal scale by extracting the mean value for all the variables and joined them with district shapefile. Other variables available in district level were also joined with district shapefile and finally all 13 variables were visualized using the QGIS software (Fig. 2). For GeoDetector analysis, the data needs to be discretized. We used the commonly applied natural breaks method to convert continuous variables into categorical variables [28, 33].

### Mapping incidence and epidemic spread rate

We visualized cumulative COVID-19 incidence rate for three different periods to represent the first, second and third waves that Nepal experienced. For this we chose a choropleth mapping approach, where the incidence was shown by bivariate color gradient. We smoothed the incidence rate following the Spatial Empirical Bayes (SEB) smoothing approach in GeoDa software to address unstable incidence rate associated with small population [34]. In addition, we also computed epidemic spread rate for all three waves following the previous work [35] and mapped the distribution patterns of the spread rate. For this, the cumulative number of COVID-19 cases was divided by the number using the following formula:

1 $${V}_{i = }\frac{{S}_{i}}{M-{N}_{i}}$$

where ${V}_{i=}$ represents epidemic spread rate in district i, ${S}_{i}$ represents the cumulative number of COVID-19 cases in region i, and M represent the three different dates used for the analyses and ${N}_{i}$ represents first confirmed COVID-19 cases in Nepal.

### Spatial autocorrelation assessment

We assessed spatial distribution patterns quantitatively using spatial autocorrelation approach. For this we computed global Moran’s I and assessed spatial dependency on the distribution of spatially smoothed incidence rate of COVID-19 over 77 districts of Nepal. The Moran’s I value ranges between − 1 (perfect dispersion) to + 1 (perfect clustering), whereas 0 indicates the random distribution. The greater the absolute value of Moran’s I, the stronger the spatial autocorrelation. The Z statistic was used to assess the statistical significance of Moran’s I Index; an absolute score larger than 1.96 coincides with a significance level at p = 0.05 and was interpreted as significant. Statistical significance was tested using randomization based on 999 permutations. Global Moran’s I is a simple translation of a nonspatial correlation measure to a spatial context. Mathematically it is expressed as

2 $$I=\frac{n\sum_{i=1}^{n}wij({x}_{i}-\overline{x })({x}_{j}-\overline{x })}{{S}^{2}\sum_{i}^{n}\,\,{\sum }_{j}^{n} {w}_{ij}}$$

where, I is the global Moran’s I index, X_i_ and X_j_ are spatially smoothed COVID-19 incidence rate, W_ij_ is the spatial weight matrix, S^2^ represents the variance, and n represents number districts.

Global Moran’s I being a global measure provides a single measure for the entire data set but cannot provide local variation in spatial autocorrelation, hence, cannot map the clustering patterns. To overcome this limitation, we utilized the Local Moran’s I statistic, commonly known as LISA (Local Indicators of Spatial Association) [36] to identify and map specific spatial autocorrelation patterns (High–High, Low–Low, High–Low, and Low–High) as well as the locations. HH can be considered spatial hotspots and LL cold spots, while the latter two categories are spatial outliers. Local Moran’s I statistics is expressed by Anselin [36] as follows:

3 $${I}_{i}=\left(x-\overline{x }\right){\sum }_{i=1 j\in ji}^{n}{w}_{ij} \left({x}_{j}-\overline{x }\right)$$

where j_i_ represents neighborhood in I region; j only the neighboring j_i_ and the $\overline{x }$ the mean of neighborhood observation. Both local and global versions of Moran’s I were computed using the GeoDa open-source software. The computation was permuted 99 times and the significance filter was set to 0.05. The first order Queen’s contiguity weight matrix was chosen to define the spatial relationship.

### GeoDetector analysis

We used GeoDetector novel spatial variance analysis approach developed by Wang et al. [37] to understand which environmental factors influences/drives the spatial patterns of COVID-19 incidence in Nepal. It is based on the concept of spatial stratified heterogeneity (SSH); referring to a within sub-region variance of less than that between the sub-regions [27]. The GeoDetector is a widely used approach in geography, environmental science, epidemiology, and other fields, where spatial analysis is crucial. The core idea of GeoDetector is that if an explanatory variable (x_1_, x_2_, …. x_n_) contributes to the response variables y, the spatial distribution between two is similar and this similarity or the spatial association can be measured by the power of q statistics. It is assumed that if environmental and socioeconomic factors have influence on the distribution of COVID-19 incidence rate in different time then the spatial distribution pattern is similar with that of COVID-19 incidence rate.

The geographical detector is composed of four subdetectors: factor detector, interaction detector, risk detector, and ecological detector. Among them, we used the factor detector and interaction detector to identify the individual influence and interactive influence of different variables on the distribution of cumulative COVID-19 incidence rate.

***Factor detector***: Factor detector is the first component of GeoDetector which assesses the influence of a potential explanatory variable on the spatial distribution of the dependent variable. It quantifies the explanatory power of variable by calculating a statistical measure called the q-statistic which is computed as follows:

4 $$q=1-\frac{1}{N\sigma 2}\sum_{h=1}^{L}(Nh\sigma 2h)$$

where σ denotes the variance of Y in the study area; N is the size of the population Y which is composed by L strata (h = 1,2,3…L) and σh2 stands for the variance within the stratum h. The value of q statistic ranges from 0 to, 1 and 0 denotes the determinate power of q-statistic. The bigger it is, the more determinant power of the factor X. If q = 1, Y is completely determined by X. On the contrary, if q = 0, the factor X is completely unrelated to Y.

***Interaction detector***: The GeoDetector can also evaluate the interaction between two factors to see if they jointly affect the spatial distribution of the dependent variable. It can determine whether the interaction between factors is independent, enhances each other, or weakens each other (Table 2). In interaction computation, first the q‐values (q(X1) and q(X2)) between two environmental factors is calculated. Then, the q‐values of the two environmental factors after interaction (q(X1 ∩ X2)) are compared with those of the individual environmental factors (q(X1) and q(X2)). Finally, the form of interplay among the two drivers is determined by comparing the distinct results.

**Table 2:** Definition of the interaction relationship in the GeoDetector model

q(X1 ∩ X2) < Min(q(X1), q(X2))	Nonlinear weaken	The interaction between two variables nonlinear weakens the influence of a single variable
Min(q(X1),q(X2)) < q(X1 ∩ X2) < Max(q(X1),q(X2))	Univariate weaken	The interaction between two variables univariate weakens the influence of a single variable
q(X1 ∩ X2) = q(X1) + q(X2)	Bivariate enhanced	The interaction between two variables bivariate enhanced the influence of a single variable
q(X1 ∩ X2) = q(X1) + q(X2)	Independent	The interaction between two variables is independent
q(X1 ∩ X2) > q(X1) + q(X2)	Nonlinear enhanced	The interaction between two variables nonlinear enhanced the influence of a single variable

We computed factor detection and interaction detection of spatially smoothed cumulative incidence rate using the GD package in R [38]. For the factor analysis, we considered all three selected time nodes, while the interaction detection was computed only for the last date of the study period.

## Results

### Spatiotemporal dynamics of COVID-19

**Fig. 3:**
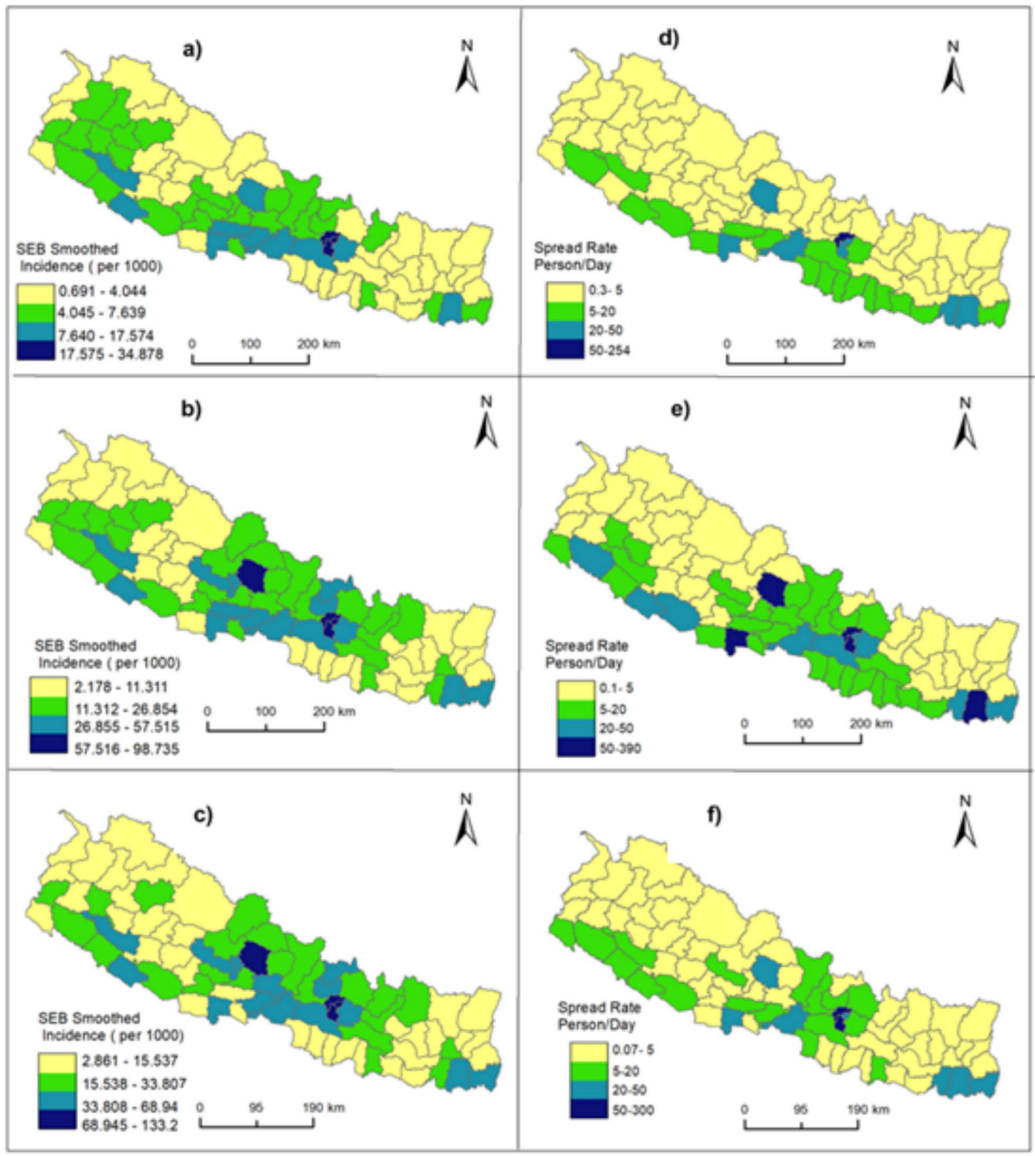
Spatially smoothed incidence rate of COVID-19 during **a** March 1, 2021; **b** November 1, 2021; **c** December 31, 2022; **d** spread rate January 23, 2020–March 1, 2021; **e** spread rate January 23, 2020–November 1, 2021; and **f** spread rate January 23, 2020–December 31, 2022

Spatial distribution of spatially smoothed COVID-19 incidence rate and its evolution is presented in Fig. 3. On March 1, 2021, the SEB smoothed cumulative incidence rate of COVID-19 ranged from 0.69 to 34.88 per 1000 population. The highest incidence rate was observed in Kathmandu, Bhaktapur and Lalitpur districts, where the SEB smoothed incidence rate was above 17.5 per 1000 population. In this period 35 districts (with yellow in Fig. 3) had the lowest incidences below 4 people per 1000 population. The cumulative incidence rate started to rise with time and reached 98.75 per 1000 population on November 1, 2021. In this time, the highest incidence was observed in 5 districts and the lowest incidence was in 31 districts of Nepal. Kathmandu, Bhaktapur, Lalitpur and Kaski were the districts with the highest incidences, where the incidence rate was above 57 persons per 1000 population. The lowest incidence was observed in the western mountain and hill districts, central Tarai districts and eastern mountain districts. On December 31, 2022, the disease further spread, and the highest incidence reached 133 persons per 1000 population. As in the second time node, the districts of Kathmandu Valley and Kaski District had the highest incidence, while the eastern mountain, western mountain and central Tarai were low incidence reported districts.

The median of epidemic spread rate was 3 person per day during all the selected time node with considerable heterogeneity in the distribution across Nepal. The highest spread rate was observed in Kathmandu District along with Bhakatapur and Lalitpur in all three selected time nodes, where the spread rate was above 50 persons per day. In Kathmandu, it was above 250 for all the time nodes. Other high spreading districts were Lalitpur, Morang, Kaski and Bhaktapur, where the spread was above 40 persons per day. The low spread, less than 5 persons per day, was observed in 51, 36, 44 districts with distributed mountain and hill district of eastern and western Nepal in the first, second and third selected time nodes, respectively.

**Fig. 4:**
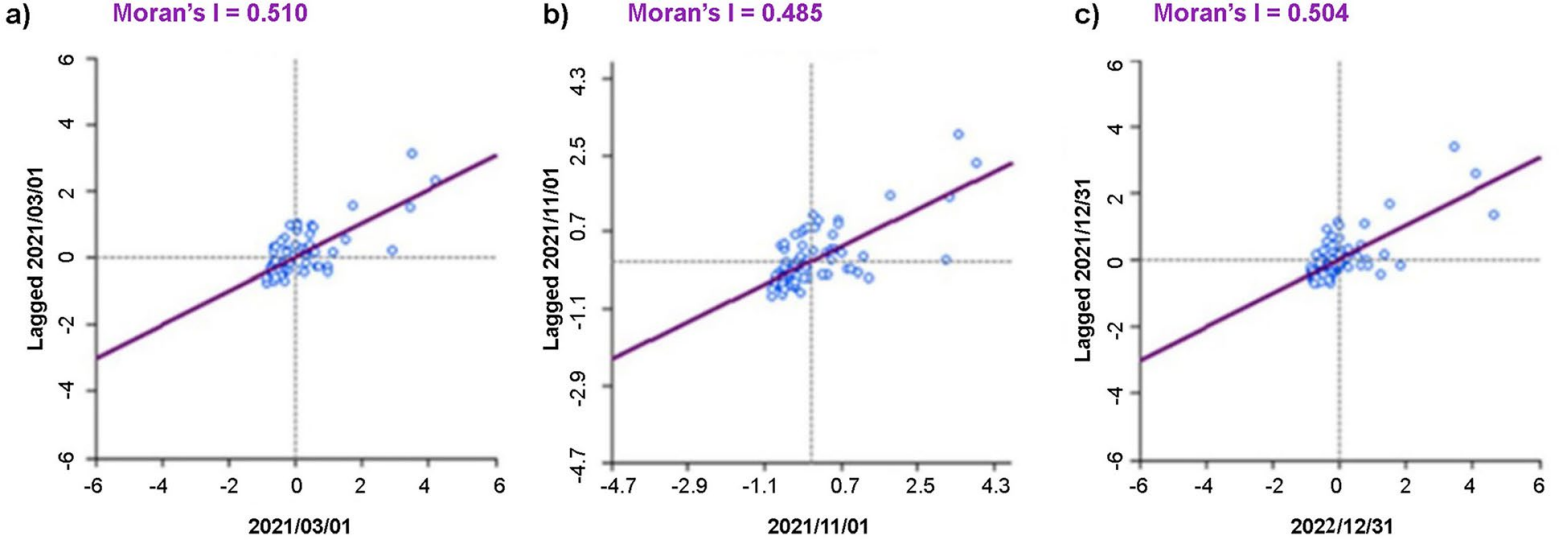
Moran’s scatter plots of spatially smoothed incidence rate of COVID-19 during **a** March 1, 2021; **b** November 1, 2021; and **c** December 30, 2022

Moran’s scatter plots (Fig. 4) show distribution of COVID-19 incidence rate of 77 districts with average incidence rate of the neighborhoods. In all the selected time nodes, the incidence rate of most of the districts were concentrated around the origin. However, a few districts’ values were in the first quadrant, far from the origin. The Moran’s I was 0.51 during the first-time node 0.49 during the second and 0.50 in the last time node, while the expected value for all three-time nodes was 0.12 (p < 0.01). No significant change was observed in Moran’s I over the time, though it was slightly lower during the second wave of COVID-19 in Nepal.

**Fig. 5:**
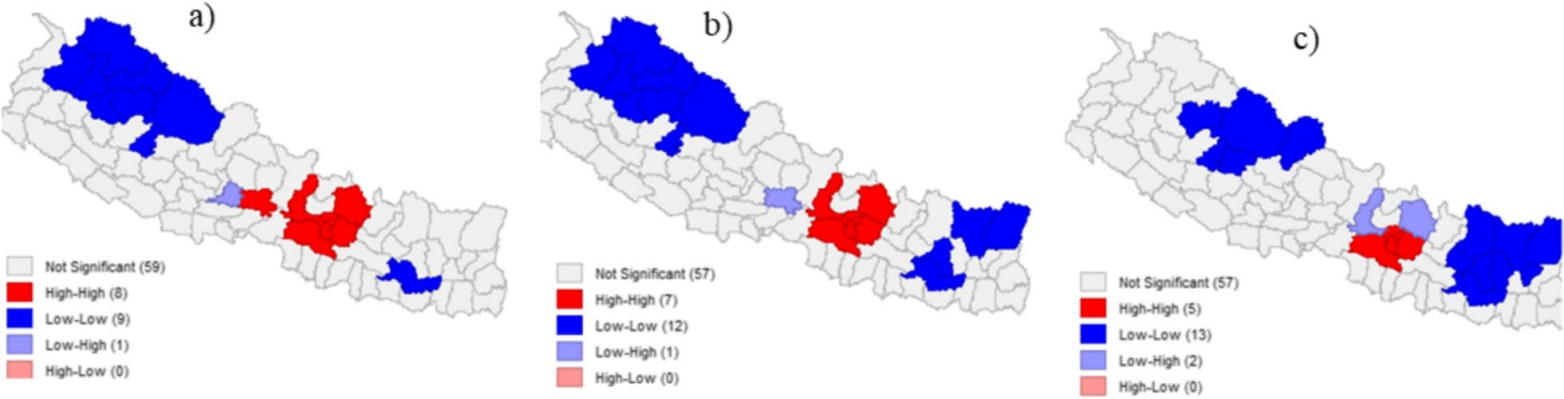
LISA MAP and Global Moran’s I SEB smoothed incidence during **a** March 1, 2021; **b** November 1, 2021; and **c** December 30, 2022

Local spatial autocorrelation analysis was subsequently performed, and the results are displayed in the form of a Local Indicators of Spatial Association (LISA) map (Fig. 5). The High–High clustering type, which is also known as hotspot, was observed in Kathmandu and neighborhood districts. There was no significant spatial shift of High–High cluster over the time, though the number of neighborhood districts was different in the different period. Similarly, Low–Low clustering, which is known as cold spots, were observed in the western and eastern mountain districts. The Low–Low clustering in the west shrunk, while it expanded in the east.

### COVID-19 incidence and driving factors

**Fig. 6:**
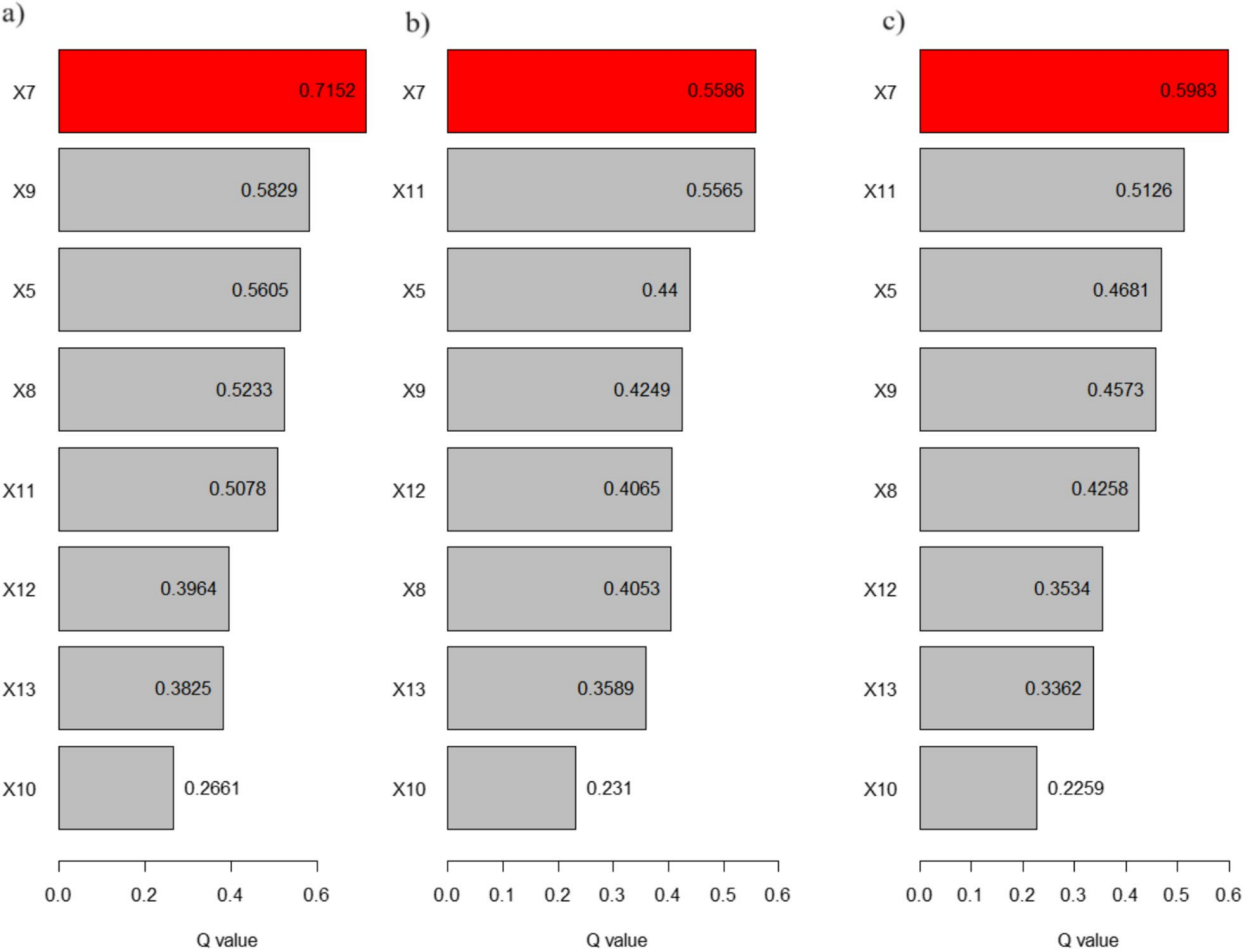
Single factor effects of different drivers (labeled as indicated in Table 1) on the spatial distribution of cumulative COVID-19 incidence rate for **a** March 1, 2021; **b** November 1, 2021; and, **c** December 30, 2022

The results of factor analysis for three selected time nodes are shown in Fig. 6. In the last time node, the distribution of road density (X7) remained most important driver to explain the spatial variation of SEB COVID-19 incidence rate in Nepal with q value 0.59 followed by distribution of ICU beds (X11) (0.51, p < 0.001) and population density (× 5) (0.46, p < 0.001). The NTL (X9), POI density (X8) and distribution of ICU bed) of the hospital were three other important drivers which can explain the variability in COVID-19 above 40% of spatial variation of the incidence rate. The explanatory power of selected variables was observed slightly higher in the first node of the pandemic. Temperature, precipitation and NDVI were statistically insignificant to explain the variability of SEB smoothed COVID-19 incidence rate in all three-time nodes.

**Fig. 7:**
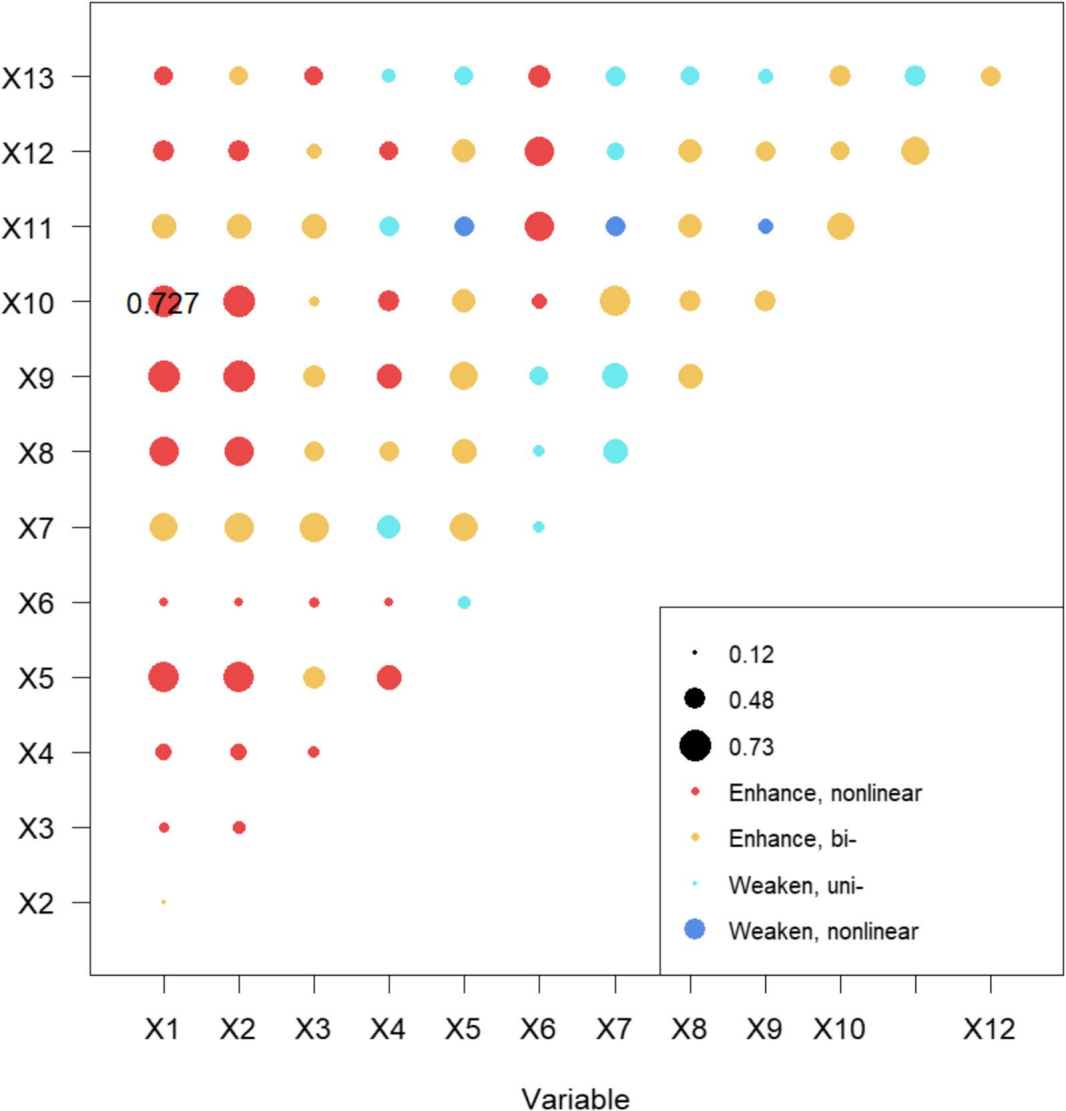
Interaction effects of selected drivers (labeled as indicated in Table 1) with the distribution of spatially smoothed COVID-19 cumulative incidence rate of December 30, 2022

We also evaluated combined effects through the interaction detector option available in GeoDetector analysis and found both enhanced and weakened effects of various drivers on the distribution of COVID-19 incidence (Fig. 7). For example, combined effects of maximum temperature and wealth were enhanced maximum nonlinearly (q value 0.72) compared to the unsignificant effects of minimum temperature and low effects (q = 0.2) of wealth. Similarly, strong enhanced nonlinear effects were observed between wealth and minimum temperature and between NTL and maximum and minimum temperature. Strong interactive effects of ICU bed and ventilator with elderly population was also observed. Other interactions among explanatory variables included enhanced bilinear and weakened bilinear.

## Discussion

This study assessed district level spatiotemporal dynamics of COVID-19 incidence rate in Nepal and associated drivers responsible for occurrence and transmission of the disease. We chose three-time nodes to capture evolution of spatiotemporal dynamics representing the first, second and third waves of COVID-19 transmission in Nepal. The results revealed uneven and heterogeneous distribution and well-developed disease clusters in all three selected time nodes during the pandemic. The GeoDetector analysis revealed that socioeconomic inequalities and heterogeneous distribution of medical resources can largely explain district level spatial variability of COVID-19 incidence rate in Nepal. To the best of our knowledge, this is the first spatial explicit study of spatiotemporal patterns of COVID-19 incidence and associated drivers in Nepal and findings of this study could be vital to inform COVID-19 intervention polices and target the health care resources optimally.

Our results showed distinct spatial variability and heterogeneity in the spatial distribution of COVID-19 incidence rate in Nepal during the first 22 months of the pandemic. The heterogeneity and spatial variation persisted continuously during the entire study period. Kathmandu remained the center of infection throughout the pandemic, although the transmission gradually spread throughout the country with the pace of time. Our results are consistent with several previous studies across the world, where spatial variation and heterogeneity were observed during different phases of the pandemic [33, 35, 39]. However, we did not observe major shifts (changes) in spatiotemporal patterns of the transmission across three major waves despite distinct characteristics in terms of dominant variants and control interventions executed by the government. The potential reasons could be lack of site specific target interventions in the country. However, aspatial study reported effects of government policies and control interventions in the reduction of transmission [40].

The GeoDetector analysis identified several important drivers influencing spatial variations and heterogeneity in the distribution of COVID-19 incidence in Nepal. Consistent with several previous studies, we found road density [31], POI density [13, 41] and NTL [42, 43] as important drivers for the heterogeneous distribution of COVID-19 incidence rate in Nepal. These factors are important socioeconomic proxies indicating places with high human activities such as urban and densely populated areas with higher probability of transmissions of COVID-19 [44]. High population density was also identified as an important drivers of COVID-19 transmission in India [45]. Similarly, population density [28, 35] and uneven distribution of medical resources [33, 35] were also influencing factors in the spatial distribution of COVID-19 incidence rate. Higher population density provides the susceptible population for the infection and frequent interpersonal contact, enhancing virus transmission in the local area and increasing infection chances [46]. Unlike the previous study in Turkey [39], proportion of elderly population was not found significant to explain spatial variation of COVID-19 incidence rate in Nepal for all three selected time nodes. The discrepancies could be due to different population composition between Nepal and Turkey. Role of environmental factors including temperature, precipitation and NDVI were statistically insignificant in the single factor analysis which is consistent with the study from India [47] and China [48] and the USA [15].

The spatial transmission dynamics of COVID-19 in Nepal remained complex across the wave influenced by not only independent drivers but also the interaction of several factors. We observed both enhanced and weakened interactions. For example, we observed significant interaction effects between maximum temperature and wealth, with the explanatory power reaching 0.72. This indicates that 72% of the spatial variation in the cumulative COVID-19 incidence rate for December 2022 can be explained by the interaction between maximum temperature and wealth. This is notably higher than the independent effects of maximum temperature (X1) and wealth (X10). A potential explanation for this is that higher temperatures in wealthier areas may lead to increased mobility, thereby facilitating the transmission of the virus. Another important interaction effect was observed between the proportion of the elderly population and the number of ICU beds. The underlying reason for this interaction could be that both factors contribute to an increased likelihood of severe COVID-19 outcomes. When combined, their impact appears to be amplified, leading to worse outcomes in affected populations. The weakened interaction was observed between road density and NTL, and road density with POI density indicating their independent effects is higher than the combined effects. Furthermore, urban environment has a greater influence in explaining the building density/population density in Chinese cities [49] that indicates how attributes of built environments can be applied in the prediction of COVID-19 [49].

The association of COVID-19 incidence may have varied over the time due to different control interventions against transmission of the virus [50]. For example, PM2.5 and mobility were not associated with COVID-19 during the lockdown but later it was assonated [51]. However, in this study, we could not find time varying explanatory power of selected drivers in explaining the distribution of COVID-19 incidence across the time node. The potential reason could be absence of place specific strategies in the control and intervention of COVID-19 during the pandemic.

This study has several policy implications. For the first time, we quantified the spatial distribution of COVID-19 incidence rates and spreading patterns across all three COVID-19 waves in Nepal, along with their associated drivers. Although previous attempts were made to characterize COVID-19 dynamics in Nepal [26, 52], those methods were aspatial and did not explore influencing factors. The GeoDetector method used in this study is a novel, spatially explicit approach based on the concept of spatially stratified heterogeneity. However, some limitations associated with this study also need to be considered. The first limitation is discretization of quantitative data which weakens the power of continuity [28]. GeoDetector is sensitive to geographic scale and spatial stratification, and differences in scale and stratification may lead to significant changes in local regions or domains [33]. Second, this district level analysis is coarse and may have masked local level spatial heterogeneity of COVID-19 incidence rate below the district level including potential heterogeneity in municipal and ward level making it less useful to inform local level health policies. We urge that future studies should be undertaken at higher resolution at least at municipal level. For this, health authority of Nepal should make health data available in higher granularity, at least at the municipal level in the context of new state restructure, where three levels of government (federal, provincial and municipal) function in Nepal. However, the district level resolution on the context of Nepal is highest granularity for the currently available health data in Nepal. In addition, the lack of human mobility data hinders our understanding of its role in explaining the spatial variability of COVID-19 incidence in Nepal.

## Conclusions

This study analyzed the spatial distribution patterns of COVID-19 incidence using the cases reported to COVID-19 national surveillance system of government of Nepal from first case until the end of December 2022, covering all three major waves in Nepal. The results revealed a heterogeneous distribution, where the centralized outbreak occurred mainly in Kathmandu and neighborhood. Despite different interventions, including the successful administration of vaccination, this study did not find any significant change in development and evolution of hotspot during the first, second and third waves of COVID-19 in Nepal. The GeoDetector-based analysis revealed distribution of medical resources such as ventilator, ICU bed and drivers related to socioeconomic inequalities such as road density, nighttime light, POI density and population density as the important factors in explaining spatial variation and heterogeneity of COVID-19 incidence rate consistently for all the time nodes. The climate and environmental factors such as NDVI, temperature and precipitation were statistically insignificant when considering individually. However, these factors enhanced the explanatory power jointly with other explanatory variables such as distribution of wealth, NTL, road density indicating a strong interactive effect and characterizing the COVID-19 transmission dynamics as complex process. The government of Nepal should prioritize targeted resource allocation, strengthen health systems in vulnerable areas, integrate socioeconomic and environmental data into public health planning and enhance disease surveillance mechanisms to address spatial inequalities and improve future pandemic preparedness.

## Data Availability

No data sets were generated or analysed during the current study.
